# Microgravity and Space Medicine 2.0

**DOI:** 10.3390/ijms23084456

**Published:** 2022-04-18

**Authors:** Daniela Grimm

**Affiliations:** 1 Department of Biomedicine, Aarhus University, Ole Worms Allé 4, 8000 Aarhus, Denmark; dgg@biomed.au.dk; Tel.: +45-2137-9702; Fax: +45-8612-8804; 2 Department of Microgravity and Translational Regenerative Medicine, University Clinic for Plastic, Aesthetic and Hand Surgery, Otto von Guericke University, Pfälzer Str. 2, 39106 Magdeburg, Germany; 3 Research Group “Magdeburger Arbeitsgemeinschaft für Forschung unter Raumfahrt-und Schwerelosigkeitsbedingungen” (MARS), Otto von Guericke University, 39106 Magdeburg, Germany

This Special Issue (SI), “Microgravity and Space Medicine 2.0”, comprises research articles on the research areas of gravitational biology, space medicine and molecular oncology. It covers studies investigating the effects of altered gravity conditions on poorly differentiated follicular thyroid cancer cells and bacteria (*Escherichia coli*) during real microgravity (r-µ*g*) on the International Space Station (ISS) [1,2]. Furthermore, it addresses the impact of simulated microgravity (s-µ*g*) on human cells and of hypergravity (hyper-*g*) on C57BL/6 mice [3]. In addition, two research articles [4,5] investigated *Euglena gracilis*, a photosynthetic flagellate, which had been studied earlier on board of the American Space Shuttle Columbia [6]. The two novel studies proposed *Euglena gracilis* as a working model of gravitaxis [4], and the applied molecular toolkits and methods can be used to bioengineer *E. gracilis* for fundamental studies in space or on Earth [5].

Space or µ*g*-research is a novel and unusual method applied to study wound healing, tissue engineering or to fight cancer, among others. Cancer researchers and space biologists decided to apply the power of µ*g* to advance translational regenerative medicine, tissue engineering or to find future cancer therapies [7,8,9].

Space travel has always been a dream of humankind. Humans live under the constant force of gravity on Earth. Life in space has enormous well-described effects on our health [10]. To counteract these health problems, a large number of studies focusing on cardiovascular changes, bone loss or the immune system have been performed in recent years [11,12,13]. A spaceflight negatively influences astronauts’ bone health, similar to mechanical unloading on Earth. Without countermeasures, bone formation and mineral deposition are decreased during a long-term stay on the ISS [14]. One contribution of this SI addresses this health problem. The authors showed that the nutraceuticals curcumin, carnosic acid and zinc synergistically promoted the process of osteogenesis in cultured 7F2 osteoblasts exposed to a random positioning machine (RPM) and mitigated inhibition of differentiation and maturation [15]. Such intermixes of phytonutrients might be tested in future space missions to investigate whether they are effective in humans against bone loss.

Differences in cell growth, gene and protein expression changes have been described in various models on Earth and in space [7,8]. Microgravity impacts survival, apoptosis, proliferation, migration, adhesion, the cytoskeleton, the extracellular matrix, focal adhesion and growth factors in human cells [7].

µ*g*-research studies as performed and published in this SI had been realized in space on the ISS or using special devices designed to create µ*g* on Earth. These ground-based facilities are acknowledged by the European Space Agency (ESA) and National Aeronautics and Space Administration (NASA) and used worldwide for µ*g*-experiments on Earth in our laboratories. Examples are the NASA-developed Rotating Wall Vessel, the two- and three-dimensional (3D) clinostat or the RPM. These machines were extensively reviewed in [7].

This SI presents nine research articles investigating the impact of r-µ*g* on cells [1] and bacteria [2] as well as of s-µ*g* on human cells [15,16,17,18]. The effects of hyper-*g* were tested using a small animal centrifuge on mice [3].

These nine excellent papers were published as detailed in Table 1.

This SI covered two publications investigating follicular thyroid cancer cells and bacteria on the ISS [1,2]. Moreover, four publications are listed which investigated changes of human cells exposed to s-µ*g* using the RPM [15,16,17,18]. The effect of hyper-*g* using a small animal centrifuge was studied in mice [3]. Additionally, two research papers investigated *Euglena gracilis* with modern molecular biological methods [4,5].

The CellBox-1 experiment studied human thyroid cancer cells (FTC-133 cell line) in an automatic hardware on the ISS (SpaceX CRS-3 cargo mission) [19,20]. Wise et al. investigated the supernatants of the space-flown FTC-133 follicular thyroid cancer cells and static controls and analyzed the exosomal microRNA composition [1]. The use of cutting-edge technologies delivered further information about the cellular changes of thyroid cancer cells in space even several years after the space mission. Furthermore, it was shown how adaptable tumor cells react to changes in the surrounding environment [1].

The second ISS experiment studied bacteria in space [2]. The authors measured changes in bacterial physiology caused by MF and r-µ*g* in *E. coli* grown in a specially developed device aboard the ISS. The detected effects of MF on *E. coli* cultivated on the ISS might be helpful for industrial purposes. The potential of MF to modify bacterial behavior in space may be useful for future space missions [2].

Osteoblastic cells were exposed to the RPM to test the effects of antioxidant nutraceuticals under s-µ*g* [15]. The authors showed that curcumin and carnosic acid and the trace element zinc promoted cellular growth in the absence of traditional osteogenic media. These three nutraceuticals stimulated osteogenic differentiation. The osteogenic effects of the plant-derived nutraceuticals were synergistic. The cells treated with the nutraceuticals could counteract RPM-based inhibition. Therefore, intermixes of phytonutrients may be interesting countermeasures against bone loss in space [15].

Another study investigated fibroblasts on the RPM and reported that fibroblast differentiation is severely impaired after RPM exposure for short periods [16]. Furthermore, fibroblasts’ conversion into myofibroblasts was inhibited. The authors demonstrated that the interplay between fibroblasts and keratinocytes in 3D co-culture experiments was remarkably altered, resulting in abnormalities in organoid-like structures. These findings may be caused by oxidative damage enacted by the stress associated with s-µ*g* [16]. In addition, a second study focused on fibroblast differentiation during microgravity [17]. The results revealed an impairment of fibroblast differentiation and a decreased matrix remodeling and production under RPM conditions. Furthermore, RNA seq data showed that RPM exposure had less effect on fibroblast transcriptomes, while s-µ*g* triggered changes in the transcriptome of myofibroblasts [17].

Zhivoderniko et al. [18] demonstrated that a 10-day RPM-exposure of MSCs induced a reduction in the collagenous components of the extracellular matrix (ECM). This result may be caused by the decrease in collagen synthesis and activation of proteases [18]. The authors showed that ECM-associated molecules of both native and osteocommitted MSCs may be involved in bone matrix reorganization during a space mission.

Little is known about the joint effect of hyper-*g* and medications on organ functions under outer space environment conditions. A further contribution to this SI investigated whether single and multiple loads of hyper-*g* stress affect APAP nephrotoxicity and hepatotoxicity in mice [3]. The authors showed that kidney function was affected when APAP was coupled with hyper-*g* stimulation. Furthermore, multiple hyper-*g* loads could ameliorate APAP toxicity via adaptation and enabled the mice to overcome kidney injury (KI). These are novel mechanistic insights explaining how hyper-*g* stress plus APAP medication might induce KI, which may be overcome by repeated hyper-*g* exposure of mice [3].

In summary, the excellent papers included in this SI report novel findings in the field of “Microgravity and Space Medicine”.

I would like to thank all the authors who supported this SI. I am convinced that the application of space research using the ISS as well as devices for s-µ*g* in combination with novel molecular biological technologies will be useful for the health protection of future astronauts, cosmonauts, taikonauts or space tourists who conquer the universe during deep space exploration missions and will also be applicable in translational regenerative medicine on Earth.

## Figures and Tables

**Table 1:** Contributions to the Special Issue “Microgravity and Space Medicine”.

Author	Title	Topics and Results	Type	Reference
Nasir, A. et al.	Molecular Cross-Talk between Gravity- and Light-Sensing Mechanisms in *Euglena gracilis*	Indication of a fine-tuned relationship between gravity sensing (gravitaxis) and light sensing in *E. gracilis* Protein kinase A (PKA) shown to be involved in phototaxis and gravitaxis The study reports the localization of the specific PKA and its relationship with PAC (photoactivated adenylyl cyclase)	Research article	[4]
Fedeli, V. et al.	Microgravity Modifies the Phenotype of Fibroblast and Promotes Remodeling of the Fibroblast–Keratinocyte Interaction in a 3D Co-Culture Model	Simulated microgravity (RPM) impairs fibroblast conversion into myofibroblast and inhibits their migratory properties The interplay between fibroblasts and keratinocytes is remarkably altered in 3D co-culture experiments Ultra-structural abnormalities and down-regulation of α-SMA that translocate in the nucleoplasm and concomitant modification of the actin–vinculin apparatus	Research article	[16]
Domnin, P.A. et al.	Combined Impact of Magnetic Force and Spaceflight Conditions on *Escherichia coli* Physiology	Changes in bacterial physiology caused by magnetic force (MF) and r-µ*g* in *Escherichia coli* grown in a specially developed device aboard the ISS r-µ*g*, r-µ*g* + MF, and MF alone induced an up-regulation of Ag43 auto-transporter and cell auto-aggregation r-µ*g* + MF induced superior up-regulation of enzymes of the methylglyoxal bypass and down-regulation of glycolysis and TCA enzymes compared with SF conditions MF strengthened the effects of µ*g* on the bacterial metabolism	Research article	[2]
Braveboy-Wagner, J. et al.	Nutraceuticals Synergistically Promote Osteogenesis in Cultured 7F2 Osteoblasts and Mitigate Inhibition of Differentiation and Maturation in Simulated Microgravity	7F2 murine osteoblasts were exposed to an RPM (6d, 21d) with/without curcumin, carnosic acid and zinc S-µ*g* enhanced cell proliferation in osteogenic medium The drugs partially reversed the inhibitory effects of s-µ*g* on alkaline phosphatase (ALP) activity No change of the RPM-induced reduction of osteogenic marker gene expression in osteogenic medium Synergistic effect of the intermix of the phytonutrients on ALP activity	Research Article	[15]
Wise, P.M. et al.	Changes in Exosomal miRNA Composition in Thyroid Cancer Cells after Prolonged Exposure to Real Microgravity in Space	CellBox-1 experiment: human thyroid cancer cells (FTC-133) flown to the ISS during the SpaceX CRS-3 cargo mission Analysis of the exosomal microRNA composition after r-µ*g* An array scan of a total of 754 miRNA targets showed more than 100 differentially expressed miRNAs Many miRNAs were implicated in thyroid disease in other studies	Research Article	[1]
Sapudom, J. et al.	Fibroblast Differentiation and Matrix Remodeling Impaired under Simulated Microgravity in 3D Cell Culture Model	Collagen-based 3D matrices to mimic interstitial tissue and to study fibroblast differentiation on the RPM Alpha-smooth muscle actin (αSMA) expression and translocation of Smad2/3 into the cell nucleus were reduced in RPM samples Impairment of fibroblast differentiation under s-µ*g* Matrix remodeling and production were decreased under s-µ*g* RPM-exposure was less effective on fibroblast transcriptomes, while it triggers changes in the transcriptome of myofibroblasts	Research Article	[17]
Becker, I. et al.	*Agrobacterium tumefaciens*-Mediated Nuclear Transformation of a Biotechnologically Important Microalga—*Euglena gracilis*	Biotechnological tools suitable for genetic modifications in the freshwater flagellate *E. gracilis* Cocultivation of *E. gracilis* and *A. tumefaciens* hosting binary vectors yielded transgenic *E. gracilis* cells expressing the marker and reporter genes The transformation was confirmed by the PCR, GUS activity and by indirect immunofluorescence assays Established ATMT system to exploit *E. gracilis* for fundamental and biotechnological studies	Research Article	[5]
Zhivodernikov, I. et al.	Simulated Microgravity Remodels Extracellular Matrix of Osteocommitted Mesenchymal Stromal Cells	Human mesenchymal stromal cells (MSCs) were exposed to the RPM for 10d Upregulation of *COL11A1*, *CTNND1*, *TIMP3* and *TNC* and down-regulation of *HAS1*, *ITGA3*, *ITGB1*, *LAMA3*, *MMP1* and *MMP11* were detected in RPM- exposed MSCs A 10-day RPM-exposure induced a decrease in the collagenous components of ECM, probably due to the decrease in collagen synthesis and activation of proteases	Research Article	[18]
Wu, H.-M. et al.	Hypergravity Load Modulates Acetaminophen Nephrotoxicity via Endoplasmic Reticulum Stress in Association with Hepatic microRNA-122 Expression	C57BL/6 mice submitted to one or three loads of +9 Gx hyper-*g* (small animal centrifuge) for 1 h with or without acetaminophen (APAP) Protein levels of cell survival markers (pAKT and pCREB) were decreased in the kidney after APAP treatment with a single hyper-*g* load Combined treatment increased kidney injury (KI) markers, serum creatinine and *Bax*, *Bcl2* and *Kim-1* transcript levels and enhanced ER stress-related markers Multiple hyper-*g* loads enabled mice to overcome KI (decreases in serum creatinine and ER stress marker levels, increased cell viability) Multiple hyper-*g* loads plus APAP elevated miR-122 levels in the kidney, originating from the liver (primary miR-122 increased only in the liver and not the kidney)	Research Article	[3]

## Data Availability

Not applicable.

## Abbreviations

APAP	Acetaminophen
D	Day
DLR	Deutsches Zentrum für Luft- und Raumfahrt
ECM	Extracellular Matrix
ESA	European Space Agency
ER	Endoplasmic Reticulum
FTC	Follicular thyroid cancer
h	hour
ISS	International Space Station
KI	Kidney injury
MF	Magnetic Force
MSCs	Mesenchymal Stromal Cells
NASA	National Aeronautics and Space Administration
r-µ*g*	Real microgravity
RPM	Random Positioning Machine
SF	Spaceflight
SI	Special Issue
s-µ*g*	Simulated microgravity
3D	Three-dimensional
